# The Role of Vortioxetine in the Treatment of Depressive Symptoms in General Hospital Psychiatry: A Case-Series and PRISMA-Compliant Systematic Review of the Literature

**DOI:** 10.3390/jcm13020531

**Published:** 2024-01-17

**Authors:** Francesco Weiss, Bruno Pacciardi, Giulia D’Alessandro, Valerio Caruso, Icro Maremmani, Stefano Pini, Giulio Perugi

**Affiliations:** 1 Psychiatric Unit 2, Department of Clinical and Experimental Medicine, University of Pisa, 56121 Pisa, Italy; francesco_weiss@libero.it (F.W.); bruno.pacciardi@gmail.com (B.P.); giulia.dalessandro26@gmail.com (G.D.); valeriocaruso79@gmail.com (V.C.); stefano.pini@med.unipi.it (S.P.); giulio.perugi@med.unipi.it (G.P.); 2 G. De Lisio Institute of Behavioural Sciences, 56121 Pisa, Italy

**Keywords:** vortioxetine, depressive symptoms, general hospital psychiatry

## Abstract

Depressive symptoms are a customary finding in hospitalized patients, particularly those who are undergoing long hospitalizations, underwent major surgical procedures or suffer from high levels of multimorbidity and frailty. The patients included in this case series shared high degrees of frailty-complexity and were evaluated within the ordinary consultation and liaison psychiatry service of the University Hospital in Pisa, Italy, from September 2021 to June 2023. Patients were administered at least one follow-up evaluation after a week and before discharge. To relate this case series to the extant literature, a comprehensive systematic review of vortioxetine safety and efficacy was performed. None of the six patients included developed serious safety issues, but one patient complained of mild-to-moderate nausea for some days after the vortioxetine introduction. Five out of six patients exhibited at least a slight clinical benefit as measured by the clinical global impression scale. Of the 858 entries screened via Scopus and Medline/PubMed, a total of 134 papers were included in our review. The present case series provides preliminary evidence for vortioxetine’s safety in this healthcare domain. The literature reviewed in this paper seems to endorse a promising safety profile and a very peculiar efficacy niche for vortioxetine in consultation and liaison psychiatry.

## 1. Introduction

Mood symptoms are highly prevalent in hospitalized patients and are often mutually associated with various medical comorbidities [1,2]. The complex polypathology of some of these inpatients poses an arduous challenge for specialists in the context of consultation and liaison (CL) psychiatry. As a matter of fact, the greater part of the available psychotropic agents entails a certain amount of risk linked to specific organ functions (e.g., heart repolarization time or haematological toxicity). Clinicians have to face a daily, more or less implicit, multivariate analysis to choose the best drugs for each particular case in terms of risk-benefit ratios [3]. Furthermore, the comorbid physical illnesses of these patients often determine pharmacokinetic and pharmacodynamic alterations that may increase the risk of side effects and/or interactions. Moreover, methods of therapeutic drug monitoring (TDM) are not available or not routinely performed for every single antidepressant [4]. The subsequent impossibility of monitoring systemic concentrations of drugs daily and assessing the pharmacokinetic phenotype represents a further major setback in CL psychopharmacology.

Vortioxetine is a relatively novel antidepressant developed by Lundbeck in 2002, with the experimental name Lu-AA21004 [5]. It was approved in September 2013 by the Food and Drug Administration (FDA) and in December 2013 by the European Medicines Agency (EMA). It is commonly classified as a serotonin modulator and stimulator (SMS) or a multimodal antidepressant. Its pharmacodynamic profile is unique, sharing a serotonin transporter (SERT) blocking activity with Selective Serotonin Reuptake Inhibitors (SSRI) and adding a host of modulatory activities on serotonin receptors (5-HT). Of primary importance among these effects is that the 5-HT1A agonist and the 5-HT3 antagonist activities are considered key in reducing the classical latency of action of most antidepressants and ameliorating cognitive symptoms [6,7]. Vortioxetine is also a 5-HT1B partial agonist, 5-HT1D antagonist and 5-HT7 antagonist. It is fairly bioavailable when administered orally (about 75%), and its terminal half-life approximately amounts to 66 h [8]. It is approved for prescription at 5 mg, 10 mg, and 20 mg daily doses, although caution should be applied in patients older than 65 for lack of data.

Vortioxetine seems a promising candidate for a favourable risk-benefit ratio in the treatment of depressive syndromes in hospitalized patients with a heavy burden of medical comorbidity [9]. A 2016 meta-analysis including 12 randomized controlled trials (RCTs) and 1508 patients showed that vortioxetine was associated with a placebo-level incidence (1.2%) of serious treatment-emergent adverse events (TEAEs) in patients older than 55 [10]. Accordingly, this meta-analysis found no rate difference between vortioxetine and placebo in crucial variables such as changes in blood pressure, heart rate, electrocardiographic (ECG) intervals, liver markers and body weight. The most common TEAE are nausea and vomiting, whose incidence resulted in more or less twice as high for all-dose vortioxetine compared to placebo. Consistently, more up-to-date review accounts report similar results as to the vortioxetine safety and tolerability [11].

Considering this favourable efficacy/tolerability profile, the apparent paucity of studies addressing the use of vortioxetine in general hospital psychiatry is quite surprising. With this work, we tried to provide an innovative contribution to the subject using a twofold strategy. First, we present a case-series sample of multimorbid inpatients hospitalized for non-psychiatric conditions whose indication for psychiatric evaluation was depressive symptoms and whom we treated with different doses of vortioxetine. Second, we have performed a systematic PRISMA-compliant review to take stock of the available information, relate our experience with the current literature and outline possible paths for future research.

## 2. Materials and Methods

### 2.1. Case Series

All of the patients included in this case series were assessed within the ordinary clinical activity of the consultation and liaison (CL) psychiatry service of the Pisa University Hospital (AOUP). Relevant first consultations were administered from September 2021 to June 2023. Patients were included considering the complexity of their general medical condition in comorbidity with depressive symptoms developed or recognized during the course of their hospitalization. Inclusion criteria were: (1) hospitalization for any medical condition; (2) two or more medical comorbidities; (3) a diagnosis of “Frailty Syndrome” according to the criteria operationalized by Fried et al. [12]; (4) a psychiatric consultation request for depressive symptoms; (5) eligibility for antidepressant treatment with vortioxetine (adult, non-pregnant patient); (6) at least one week of observation after vortioxetine introduction; (7) vortioxetine dose titrated to at least 5 mg per day; (8) no other antidepressant in combination (other classes of psychotropics were allowed).

Information about patients (personal data, diagnoses, treatments, course, and discharge) was collected from the ordinary clinical documentation (medical records and discharge letter) produced during their hospitalization, in line with the good clinical practice guidelines of the AOUP. All available data about medical diagnoses, surgical and pharmacological treatment, organ insufficiencies, blood testing, instrumental examinations and other specialistic consultations were collected upon the first psychiatric consultation. A complete psychiatric examination was performed, a clinical global impression (CGI) Field [13] severity scores were recorded, and psychotropic treatment was introduced. All patients received at least one further evaluation before discharge, during which CGI severity, CGI improvement and CGI efficacy index were filled out. Complications, possible TEAEs, and mental and physical conditions at discharge were documented in the clinical diary and discharge letter. The patient had expressed their consent to the use of anonymized clinical data for research purposes at the moment of their admission.

### 2.2. Systematic Review of the Literature

**Figure 1:**
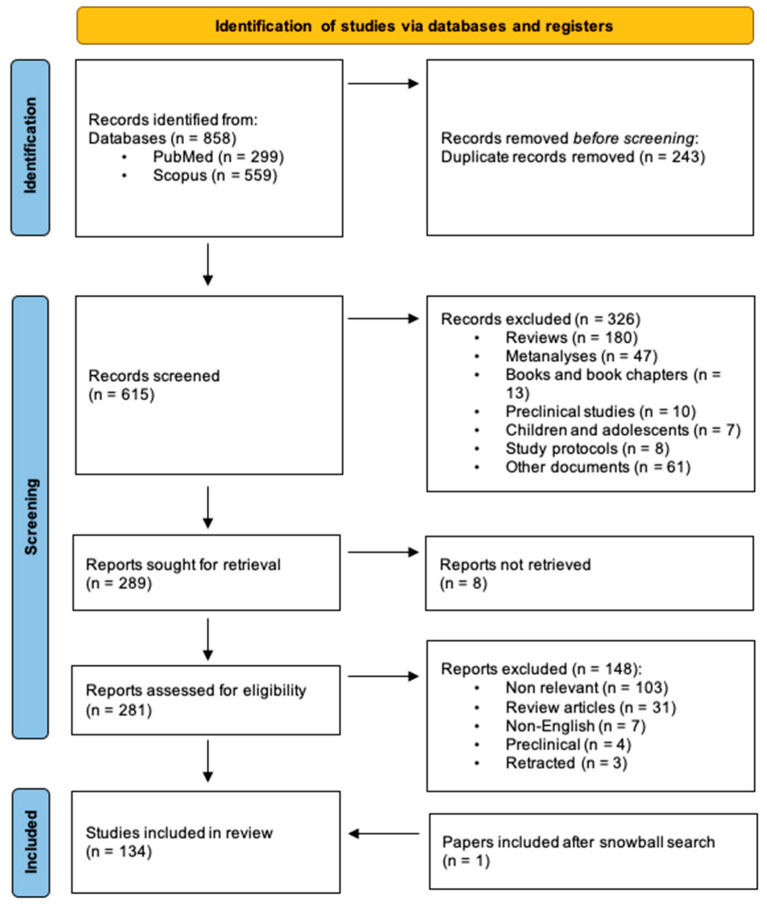
Identification of studies via databases and registers.

The systematic review was performed according to the Preferred Reporting Items for Systematic Reviews and Meta-Analyses (PRISMA) statement [14,15,16]. Search methods and results are highlighted in Figure 1.

First, a comprehensive literature search on PubMed and Scopus was performed from inception to 16 June 2023, cross-checking the obtained references. The systematic search was conducted using the following search string: “Vortioxetine AND (liver disease OR hepatic disease OR hepatopathy) or vortioxetine AND (kidney disease OR renal disease OR nephropathy) OR vortioxetine AND (heart disease OR cardiovascular disease OR cardiopathy) OR vortioxetine AND (brain disease OR encephalopathy OR stroke OR dementia OR neurodegeneration) OR vortioxetine AND (inpatients OR hospital OR medical condition) or vortioxetine AND (safety OR tolerability OR adverse)”.

We included all studies exploring the efficacy, safety, and tolerability of vortioxetine that supply original data. Taking as a reference the classification of clinical research proposed by Grimes and Schulz [17], we set the following inclusion criteria: descriptive observational studies (case series and case reports), analytical observational studies (cross-sectional, case–control and cohort studies), experimental studies (including open, single-blind, double-blind randomized, non-randomized, controlled and non-controlled studies). We concurrently posited the following exclusion criteria: non-English articles; studies recruiting subjects under the age of 18 years; review articles (including narrative and systematic reviews, pooled analyses, and meta-analyses); preclinical studies; study protocols; books and book chapters; expert opinions or consensus articles; other documents not exhibiting original data.

## 3. Results

### 3.1. Case Series

Six patients were included in this case series, four males and two females, aged between 64 and 77 years (arithmetic mean: 71.67 years). The main comorbidities and surgical procedures for each single patient are listed in Table 1.

Vortioxetine dose ranged from 5 to 10 mg per day, with four patients taking 10 mg/day and two patients taking 5 mg/day. Psychotropic (including opioids) and non-psychotropic co-prescribed medications for each patient are listed in Table 2.

The severity of depressive symptoms at baseline assessed with CGI severity scale ranged from 3 (“mildly ill”) to 5 (“markedly ill”). The CGI-Severity scores at follow-up resulted in a sensitive reduction, ranging from 2 (“borderline mentally ill”) to 4 (“moderately ill”). At discharge, CGI improvement scores were as follows: four patients were scored 2 (“much improved”), one was scored 3 (“minimally improved”), and one was scored 4 (“unchanged”). Considering the CGI efficacy index calculated at discharge, one patient scored 1 (“complete or nearly remission of symptoms with no side effects”), three patients scored 5 (“partial remission of symptoms with no side effects”), one patient scored 9 (“slight improvement with no side effects”), and one patient was scored 13 (“unchanged or worse with no side effects”). One safety issue was recorded (nausea in patient 06). Four patients were discharged at home, one transferred to a palliative care facility, and one transferred to an intermediate care facility. Efficacy and safety data are synthesized in Table 3.

### 3.2. Systematic Review of the Literature

The literature research with the above-presented string yielded a total of 858 articles, of which 299 were listed on PubMed and 559 were found on Scopus. After accurate duplicate screening, 243 duplicates were found and excluded. Of the remaining 615 records, 326 were excluded according to a priori exclusion criteria after abstract screening. Primary screening was performed manually, as no automation tool was employed. Excluded items were listed and subdivided into Reviews (n = 180), Meta-analyses (n = 47), Books and book chapters (n = 13), Preclinical studies (n = 10), Children and adolescents (n = 7), Study protocols (n = 8) and Other documents (n = 61). We sought to retrieve 289 papers, and we did not manage to retrieve eight entries. The resulting 281 articles underwent eligibility assessment via full-text examination, following which 148 articles were further excluded, and 134 studies were finally included in this review. Excluded items from eligibility assessment were listed and subdivided in: Non relevant (n = 103), Review articles (n = 31), non-English articles (n = 7), Preclinical (n = 4), Retracted (n = 3). Among the included articles, 72 were experimental studies, 45 were analytical observational studies, and 16 were descriptive observational studies. Open-label extension studies articles (n = 4) were conventionally included within experimental studies. Following snowballing research, we finally added a further article in the experimental studies category (n = 73), and the total number of entries included in the review reached n = 134. Since the main purpose of this work was to assess safety and tolerability issues, efficacy-related data will only be briefly summarized in the text, while safety-related findings will be exhibited in Table 4, Table 5, Table 6, Table 7 and Table 8 in an orderly manner.

#### 3.2.1. Experimental Studies

A total of 73 records were included in this macro-category. Among these, 48 studies dealt with the efficacy and tolerability of vortioxetine in the treatment of major depressive disorder. For analytical purposes, these studies were subdivided into (1) placebo-controlled studies without active reference and (2) controlled trials with active reference. Placebo-controlled studies without active reference (n = 25) were conducted starting in 2007. Among the studies (n = 12) that investigated the acute response to vortioxetine in major depression (6-, 8- and 12-week designs), four trials found a statistically significant advantage compared to placebo [18,19,20,21], two showed mixed results [22,23], and six provided no statistical difference between treatment and placebo [24,25,26,27,28], including one trial involving depressed patients with Alzheimer’s disease [29]. Three post hoc analyses (n = 3) based on one positive trial conducted in Japan found (a) that patients obtaining an early partial improvement with vortioxetine were more likely to be responders/remitters at 8 weeks [30]; (b) that vortioxetine was effective also on anxiety and anhedonic symptoms [31,32].

Efficacy data (no difference from placebo) of two further studies (n = 2) were not relevant to our review (celecoxib-placebo randomization as an “add-on” to open-label vortioxetine) [33,34], but one of the two showed that cross-titration switching to vortioxetine from other antidepressants is usually well-tolerated [34]. Among studies devoted to long-term treatment with vortioxetine (n = 8), randomized controlled studies (n = 3) exhibited favourable results in depressive relapse prevention [35,36,37]. Consistently, open-label extension studies demonstrated (n = 5) a clear clinical benefit in prolonging vortioxetine treatment after short-term response both in terms of symptoms amelioration (including anhedonia) and functional recovery [38,39,40,41,42].

Controlled trials with active reference (n = 23) were conducted starting from 2006, 21 (plus an “Erratum”) with an acute efficacy/safety design (<12 weeks). Some of these studies (n = 5) found vortioxetine to be superior in mood symptom reduction than the active comparator, including agomelatine [43,44], venlafaxine [45], desvenlafaxine [46] and duloxetine [47]. Other experiences (n = 4) found vortioxetine to be more effective in cognitive symptom reduction with respect to escitalopram, paroxetine, and duloxetine [48,49,50,51]. Half of these trials (n = 11) found no significant difference between vortioxetine compared to sertraline [52], venlafaxine [53,54], desvenlafaxine [55,56], escitalopram [55,56,57,58], vilazodone [57], and duloxetine [59,60,61,62], although duloxetine tended to perform better in these studies. Finally, a single study (n = 1) found low-dose (2.5 mg and 5 mg) vortioxetine significantly inferior to duloxetine 30 mg/day [63]. Lastly, we found a long-term (12 months) quasi-experimental investigation involving patients with mild Alzheimer’s disease, demonstrating that vortioxetine was more effective than escitalopram, paroxetine, bupropion, venlafaxine and sertraline (altogether considered) the amelioration of cognitive performances [64].

Other studies focused on functional outcomes rather than categorial diagnoses (n = 8) and are summarized in Table 4 [65,66,67,68,69,70,71,72].

**Table 4:** Studies evaluating functional outcomes.

Study	Functional Outcome	Clinimetric Tools	Results
Theunissen et al., 2013 [66]	Actual driving Cognitive performance Psychomotor performance	Actual driving test (SDLP) Divided attention task Psychomotor vigilance task	MIRT significantly impairs driving on day 2 but not on day 16 and prolongs reaction times in psychomotor vigilance and divided attention tasks compared to VOR and PLA.
Jacobsen et al., 2015 [72]	TESD	CSFQ-14	VOR: significantly associated with greater improvement in sexual function in previously SSRI-treated patients compared to ESC.
Chokka et al., 2019 [69]	Cognitive performance, workplace functioning, perceived disability	DSST, PDQ-D-20, WLQ, WPAI, SDS	VOR significantly improved outcomes in all clinimetric scales at 52 weeks.
Chokka et al., 2019 [70]	Cognitive performance, workplace functioning, perceived disability	DSST, PDQ-D-20, WLQ, WPAI, SDS	VOR significantly improved outcomes in all clinimetric scales at 12 weeks.
Jacobsen et al., 2019 [65]	TESD	CSFQ-14	PAR: significantly higher incidence of TESD compared to VOR and PLA VOR: not significantly higher incidence of TESD compared to PLA.
Jacobsen et al., 2019 [68]	TESD	CSFQ-14	VOR: significantly associated with greater improvement in sexual function in previously SSRI-treated patients compared to ESC.
Nierenberg et al., 2019 [71]	Cognitive performance	DSST	Both SSRI and VOR improved cognitive performance. VOR did not significantly outperformed standard SSRIs.
Lenze et al., 2020 [67]	Cognitive training augmentation	NIH Toolbox Cognition Battery Fluid Cognition Composite	Cognitive training plus VOR significantly improves performance compared to cognitive training plus PLA.

TESD: treatment-emergent sexual dysfunction; CSFQ-14 Changes in Sexual Functioning Questionnaire Short-Form; PAR: paroxetine; VOR: vortioxetine; PLA: placebo; SDLP: standard deviation of lateral position; MIRT: mirtazapine; SSRI: selective serotonin reuptake inhibitor; ESC: escitalopram; DSST: Digit Symbol Substitution Test; PDQ-D-20: 20-item Perceived Deficits Questionnaire for Depression; WLQ: Work Limitations Questionnaire; WPAI: Work Productivity and Activity Impairment; SDS: Sheehan Disability Scale.

A number of trials dealt with the effectiveness of vortioxetine in diagnoses other than depression (n = 10). Vortioxetine was found effective in the treatment of negative symptoms of schizophrenia [73], burning mouth syndrome [74] and irritable bowel syndrome [75] and ineffective in binge eating disorder (BED) [76] and attention deficit hyperactivity disorder (ADHD) [77]. Moreover, among studies of vortioxetine in generalized anxiety disorder (GAD) (n = 5), three 8-week trials exhibited negative results [78,79,80], while 8-week and 20-week trials showed a significant benefit over placebo [81,82]. Key information derived from experimental pharmacokinetics studies (n = 7) is summarized in Table 5 [83,84,85,86,87,88,89].

**Table 5:** Experimental pharmacokinetic studies.

Study	Half-Life (Hours)	Tmax (Hours)	Observations
Chen et al., 2013 [89]	/	/	Vortioxetine has no effect on drug metabolism catalyzed by CYP2D6, CYP2C19 and CYP3A4. Vortioxetine metabolism is influenced by CYP2D6 inhibitors and CYP3A4 inducers.
Wang et al., 2013 [88]	58.58 (10 mg) 56.41 (40 mg)	8.10 (10 mg) 8.10 (40 mg)	Vortioxetine dosed 10 mg and 40 mg has no clinically significant effect on corrected QT.
Chen et al., 2015 [87]	/	/	Vortioxetine dosed 10 mg has no effect on aspirin or warfarin pharmacokinetics.
Wilson et al., 2015 [86]	/	/	Vortioxetine effects on sleep resemble those of paroxetine: reduced total sleep time, reduced total REM sleep, prolonged REM onset latency, and increased stage 1 sleep.
Chen et al., 2016 [85]	/	/	Vortioxetine has no effect on the pharmacokinetics of ethanol, diazepam and lithium and does not impact psychomotor performance compared with ethanol or diazepam alone.
Matsuno et al., 2018 [84]	69.4 (5 mg) 66.0 (10 mg) 55.1 (20 mg) 56.6 (40 mg)	10.0 (5 mg) 10.0 10 mg) 9.0 (20 mg) 6.3 (40 mg)	Vortioxetine metabolism does not differ between sexes. Vortioxetine exposure tends to be higher in the elderly. No food effect on the pharmacokinetics of vortioxetine.
Chen et al., 2018 [83]	/	/	Vortioxetine exposure tends to be about 25% higher in the elderly, blacks and females. Severe hepatic or renal impairment does not significantly alter vortioxetine disposition.

The most interesting findings were related to vortioxetine’s safety and tolerability in comparison with other agents. Throughout all of the experimental studies reviewed here, the most commonly reported TEAE during vortioxetine treatment was nausea, whose frequency was often significantly higher than under placebo. Other very commonly reported TEAEs were diarrhoea, headache, dry mouth, drowsiness, fatigue, dizziness, and insomnia, but their frequencies were often not significantly higher than under placebo. Vortioxetine was generally well-tolerated, with a tolerability profile at least equal or superior compared to other antidepressants and only mild-to-moderate adverse events. Vortioxetine was very rarely associated with severe adverse events in all studies, and severe adverse events were unlikely to be related to medication. One of the most iterated findings was that vortioxetine does not impair sexual function compared to placebo, whereas other serotonergic or dual antidepressants consistently did so. Table 6 summarizes data extracted from randomized trials reporting adverse events-related inferential statistics.

**Table 6:** Randomized studies (including both active-referenced and placebo-control studies) report inferential statistics relative to safety data.

Study	Comparator	Significant TEAEs (Vortioxetine vs. Comparator)	Non-Significant TEAEs
Alvarez et al., 2012 [54]	Placebo	Nausea, Vomiting, Hyperhidrosis.	Headache, Dry mouth, Diarrhoea, Dizziness, Nasopharyngitis, Fatigue, Insomnia, Constipation, Blurred Vision, Anorgasmia.
Baldwin et al., 2012 [62]	Placebo	Nausea.	Headache, Diarrhoea, Vomiting, Dizziness, Dry mouth, Somnolence, Nasopharyngitis, Constipation, Fatigue, Hyperhidrosis, Insomnia, Decreased appetite.
Boulenger et al., 2012 [37]	Placebo	Nausea.	Headache, Nasopharyngitis, Dizziness, Dry mouth, Insomnia, Fatigue, Gastroenteritis.
Katona et al., 2012 [61]	Placebo	Nausea.	Headache, Dizziness, Fatigue, Constipation, Dry mouth Diarrhoea, Decreased appetite, Hyperhidrosis.
Boulenger et al., 2014 [60]	Placebo	Nausea, Dry mouth.	Headache, Diarrhoea, Dry mouth, Dizziness, Fatigue, Hyperhidrosis.
Wang et al., 2015 [53]	Venlafaxine	(Higher in venlafaxine: decreased appetite, insomnia)	Nausea, Dizziness, Headache, Dry mouth, Accidental overdose, Decreased appetite, Constipation.
Liebowitz et al., 2017 [22]	Placebo	Nausea.	Anxiety, Depression, Irritability, Decreased appetite, Difficulty achieving orgasm, Headache, Increased appetite, Increased sleep, Insomnia, Migraine, Headache, Tingling, Tiredness, Constipation, Diarrhoea.
Borhannejad et al., 2020 [52]	Sertraline	/	/

Finally, Table 7 gathers differences in withdrawal rates due to adverse events between vortioxetine and other antidepressant agents.

**Table 7:** Discontinuations due to adverse events in head-to-head trials.

Study	Participants	Vortioxetine	Comparator 1	Comparator 2
Shin et al., 2023 [56]	Vortioxetine = 42 Escitalopram = 42 Desvenlafaxine = 40	N = 1 (Vomiting = 1)	Escitalopram N = 2	Desvenlafaxine N = 3
Santi et al., 2023 [57]	Vortioxetine = 20 Escitalopram = 18 Vilazodone = 18	N = 0	Escitalopram N = 0	Vilazodone N = 0
McIntyre et al., 2023 [46]	Vortioxetine = 309 Desvenlafaxine = 293	N = 6	Desvenlafaxine N = 3	/
Lee et al., 2022 [55]	Vortioxetine = 40 Escitalopram = 43 Desvenlafaxine = 38	N = 2 (Vomiting = 1) (Fatigue = 1)	Escitalopram N = 3	Desvenlafaxine N = 2
Borhannejad et al., 2020 [52]	Vortioxetine = 30 Sertraline = 30	N = 0	Sertraline N = 0	/
Levada et al., 2019 [49]	Vortioxetine = 41 Escitalopram = 25	N = 0	Escitalopram N = 0	/
Vieta et al., 2018 [58]	Vortioxetine = 50 Escitalopram = 49	N = 3 (Not available)	Escitalopram N = 1	/
Baune et al., 2018 [50]	Vortioxetine = 48 Paroxetine = 54 Placebo = 48	N = 3 (Nausea = 2)	Paroxetine N = 3 (2 serious adverse events)	Placebo N = 1
Mahableshwarkar et al., 2015 [59]	Vortioxetine = 301 Duloxetine = 152 Placebo = 161	N = 28 (Most common: nausea)	Duloxetine N = 10	Placebo N = 4
Mahableshwarkar et al., 2015 [51]	Vortioxetine = 198 Duloxetine = 210 Placebo = 194	N = 7	Duloxetine N = 13	Placebo N = 7
Wang et al., 2015 [53]	Vortioxetine = 211 Venlafaxine = 226	N = 14	Venlafaxine N = 32	/
Boulenger et al., 2014 [60]	Vortioxetine = 302 Duloxetine = 147 Placebo = 158	N = 27 * (Most common: nausea)	Duloxetine N = 7	Placebo N = 7
Montgomery et al., 2014 [44]	Vortioxetine = 253 Agomelatine = 246	N = 15 (Most common: nausea and vomiting)	Agomelatine N = 23 (Most common: dizziness and headache)	/
Alvarez et al., 2012 [54]	Vortioxetine = 208 Venlafaxine = 113 Placebo = 105	N = 10 (Not available)	Venlafaxine * N = 16	Placebo N = 4
Katona et al., 2012 [61]	Vortioxetine = 156 Duloxetine = 151 Placebo = 145	N = 10 (Most common: nausea)	Duloxetine N = 15	Placebo N = 6
Baldwin et al., 2012 [62]	Vortioxetine = 463 Duloxetine = 155 Placebo = 148	N = 43	Duloxetine * N = 19	Placebo N = 12

* Difference from placebo reached statistical significance.

#### 3.2.2. Analytical Observational Studies

A total of 45 records were included in this macro-category. The greatest part (n = 18) were studies related to various clinical outcomes in patients suffering from major depressive disorder. All of these studies reported high levels of safety and tolerability, with no unexpected TEAEs. A number of experiences demonstrated that vortioxetine was associated with significant improvements in affective, cognitive and functional outcomes in patients diagnosed with depression [90,91,92,93,94]. RELIEVE was an international (United States, Canada, Italy, France and, independently, China) observational, prospective 24-week study protocol involving adult patients with a diagnosis of major depression initiating treatment with vortioxetine in the context of routine clinical practice. The articles derived from this design (n = 6) are reports of significantly favourable functional outcomes (Digit Symbol Substitution Test, EuroQoL 5 dimensions five levels utility index, 5-item Perceived Deficits Questionnaire-Depression, 9-item Patient Health Questionnaire, Sheehan Disability Scale) in patients after twelve (eight in the Chinese study) and twenty-four weeks of treatment with vortioxetine [95,96,97,98,99,100].

Two studies dealt with outcomes related to coronavirus disease 2019 (COVID-19): one showed that vortioxetine treatment in patients with post-COVID-19 major depression was associated with a significant reduction in depressive, cognitive and physical symptoms after three months [101]; the other reported data suggesting that vortioxetine is associated with a significantly greater reduction in depressive symptoms worsening after a COVID-19-related trauma compared to sertraline and trazodone [102]. An Italian study demonstrated that vortioxetine was associated with significantly higher therapeutic adherence relative to SSRIs and dual selective antidepressants (among the latter, the lowest risk of poor adherence was observed with sertraline and the highest with venlafaxine) [103]. A South Korean study with 3263 patients showed that improvement with vortioxetine treatment was associated with significant reductions in mood and cognitive symptoms after eight and twenty-four weeks, without any detrimental influence of age and liver dysfunction on safety and efficacy [104]. Vortioxetine was also associated with equal effectiveness in depression comorbid with GAD or alcohol use disorder [105,106]. Finally, a study reported data suggesting that vortioxetine adjunct to SSRIs is associated with a significant amelioration of depressive symptoms in patients resistant to SSRIs alone [107].

A group of studies dealt with vortioxetine use in psychiatric diagnoses other than unipolar depression (n = 7). These articles report encouraging results in the treatment of negative, cognitive and affective symptoms of schizophrenia [108,109,110], along with preliminary effectiveness in bipolar depression [111], burning mouth syndrome [112], postmenopausal depression [113], and panic disorder [114]. A further group of studies (n = 5) demonstrated that vortioxetine treatment is associated with affective and cognitive symptoms amelioration in patients with neurological conditions, including neurocognitive decline [115,116,117], Parkinson’s disease [118], and post-stroke depression [119]. Interestingly, three studies report data suggesting that vortioxetine could be an effective strategy to reduce SSRI-induced emotional blunting [120,121,122]. Finally, a large number of entries (n = 12) were pharmacovigilance studies or studies whose primary endpoints were safety/tolerability outcomes (main findings summarized in Table 8) [123,124,125,126,127,128,129,130,131,132,133,134].

**Table 8:** Summary of observational studies concerning safety and tolerability.

Study	Aim	Observation 1	Observation 2
Hughes et al., 2017 [126]	Screening of online reviews of antidepressant users and user satisfaction as a marker of drug acceptability.	VOR was associated with a higher frequency of itch, nausea, anxiety, agitation, crying, and headache compared to DUL and ESC.	VOR was associated with a lower frequency of insomnia, sexual complains, emotional numbing, and motor disorders compared to DUL and ESC.
Greenblatt et al., 2018 [125]	Comparison of VOR pharmacokinetics between obese subjects and control.	VOR accumulation and washout half-lives were significantly longer (about 50%) in obese individuals.	Possible pharmacodynamic interaction (serotonin toxicity) after VOR withdrawal might last longer than expected in obese individuals.
Mazhar et al., 2019 [133]	FAERS database vigilance study on antidepressants risk of hyponatremia.	VOR was significantly associated with a higher risk of hyponatremia (although probably overestimated).	Relative to VOR, the risk of hyponatremia was higher for CLO, TRI, MIRT, SSRIs and SNRIs.
Miao et al., 2019 [132]	Pharmacokinetics and safety of VOR in Chinese subjects compared to non-Chinese.	VOR exposure was higher in the Chinese than non-Chinese population, but difference did not reach significance.	Commonest side effects in 124 Chinese subjects were nausea (20.2%), dizziness (9.7%), dry mouth (5.6%), hyporexia (4%) and Diarrhoea (3.2%).
Woroń et al., 2019 [134]	66 cases of interaction between antidepressants and cardiovascular medications.	VOR linked to a case of hyponatremia (+HCTZ) and a case of nose/urinary bleeding (+WARF).	Commonest interaction: bradycardia (SSRI + beta-blockers) and limb swelling (SSRI + amlodipine).
Bordet et al., 2020 [130]	QT prolongation: VOR vs. SSRIs.	The association between VOR and QT prolongation was non-significant.	VOR bears a lower risk of inducing QT prolongation than SSRIs.
Eugene., 2020 [131]	Association between 30 antidepressants and somnolence.	VOR showed the lowest risk of somnolence (but no risk for levomilnacipran).	Amoxapine showed the greatest association with somnolence, probably due to highest 5HT2_A_ affinity.
Revet et al., 2020 [129]	Antidepressants and movement disorders.	VOR is significantly associated with bruxism (OR 4.71, 2.88–8.80).	SSRIs are associated with a higher risk of movement disorders compared to first-generation antidepressants.
Seifert et al., 2021 [128]	Psychotropic drug-induced hyponatremia	Out of 501 patients on VOR, 1 case of hyponatremia was registered.	Highest rates of hyponatremia were reported with CIT, VEN and ESC.
Ekhart et al., 2022 [123]	VOR TEAE reporting pattern and comparison with SSRIs.	Most frequent TEAE reported with VOR were nausea, vomiting, Diarrhoea and hypersensitivity (pruritus, rash).	Withdrawal syndrome, hyponatremia, tremor and paresthesia less often reported for VOR than SSRIs.
Healy et al., 2022 [127]	Adverse visual effects linked to antidepressants.	A case of blurred vision still present 26 days after a single VOR dose.	Most reports of persisting visual disorders involved SER, ESC, VEN, and FLU.
Quilichini et al., 2022 [124]	Withdrawal syndrome risk with different antidepressants.	VOR and AGO have the lowest risk of withdrawal syndrome.	PAR, DES, VEN, and DUL have the highest risk of withdrawal syndrome.

TEAE: treatment-emergent adverse events; VOR: vortioxetine; SSRI: selective serotonin-reuptake inhibitor; AGO: agomelatine; PAR: paroxetine; DES: desvenlafaxine; VEN: venlafaxine; DUL: duloxetine; SER: sertraline; ESC: escitalopram; FLU: fluoxetine. CIT: citalopram; AD: antidepressants. 5HT2_A_: 5-hydroxytryptamine receptor 2_A_; MDD: major depressive disorder; FAERS: Food and Drug Administration (FDA) Adverse Event Reporting System; CLO: clomipramine; TRI: trimipramine; MIRT: mirtazapine; HCTZ: hydrochlorthiazide; WARF: warfarin.

#### 3.2.3. Descriptive Observational Studies

A total of 16 records were included in this macro-category. Some of these (n = 7) include a case of hyperprolactinemia/galactorrhea [135], three cases of moderate-to-severe allergic skin reactions with petechial and pruritic features [136,137], a case of ingravescent acneiform eruption [138], a case of tinnitus [139], a case of acutely arising hemoptysis [140], and a case of restless leg syndrome [141]. There was also a report relating to a case of Call-Fleming syndrome or Reversible Cerebral Vasoconstriction Syndrome (RCVS), which arose in a twenty-year-old woman seven weeks after vortioxetine introduction. The presentation was somewhat aspecific, with features of meningism (headache, photophobia), stroke-like motor deficits (hemiparesis and dysarthria) and intracranial hypertension (projectile vomiting) and completely resolved in three weeks with vortioxetine discontinuation and introduction of nimodipine 120 mg/day [142]. One report refers to two cases of Meige syndrome (bilateral orofacial dystonia with perioral dyskinesia and blepharospasm), which occurred in two sisters, both ten days after vortioxetine introduction and resolved a few days after vortioxetine discontinuation [143]. Vortioxetine was also associated with two cases of manic switch that occurred after vortioxetine introduction (10 mg/day) in two female patients previously diagnosed with unipolar depression [144,145]. One of the two patients had a history of multiple antidepressant treatments (escitalopram, fluoxetine, paroxetine, and duloxetine) without evidence of former manic reactions.

Interestingly, for our purposes, vortioxetine was found safe in the case of a suicidal attempt by overdosage (250 mg), with no changes in blood pressure, electrocardiogram, creatinine and only modest changes in g-glutamyl-transferase levels (71 U/L twenty-four hours after ingestion) [146]. A case series showed that vortioxetine (10–20 mg/day) was safe and effective in a sample of nine patients suffering from epilepsy (seven cases were structural, and two were cryptogenetic) without any apparent destabilization of epileptic thresholds [147]. A report found a favourable outcome after three months 10 mg/day vortioxetine treatment in a patient with rapid eye movement (REM) sleep behaviour disorder previously treated with paroxetine and melatonin without benefit [148]. Another paper described a case of severe vortioxetine-induced nausea successfully treated with mirtazapine in a patient with a history of multiple SSRI trials interrupted for sexual dysfunction. The combination of vortioxetine-mirtazapine was well-tolerated, and the patient achieved significant remission of mood, anxiety and sleep symptoms without any sexual complain [149]. Finally, a paper presented a case of fatally complicated hyponatremia in a 72 years old woman who had had previous episodes of severe hyponatremia with other psychotropics (fluoxetine, amitriptyline, mirtazapine and asenapine) [150].

## 4. Discussion

In this case, report, five out of six patients displayed some improvement in depressive symptoms and only one patient resulted to be unchanged. Among them, none of the subjects suffered from clinically significant TEAE after vortioxetine introduction. Vortioxetine treatment was associated with no safety issues and nausea was the only tolerability issue recorded (in patient 06). The severity of this TEAE was mild and did not entail antidepressant switch or discontinuation. All of the patients included had different and significant physical conditions that required hospitalization, but none of these conditions worsened during antidepressant treatment nor impairment of any organ system function was evidenced at follow up. This datum is even more captivating if considered in light of the significant level of frailty shared by the patients of our sample. Intriguingly, we observed a relatively short latency of therapeutic effects, consistently with preclinical evidence [151,152,153]. To the best of our knowledge there are no previous studies concerning vortioxetine use in patients hospitalized for general medical conditions. Our clinical experience seems to be in line with the available evidence regarding vortioxetine tolerability and provides preliminary support for a safe employment of vortioxetine in hospitalized patients with a significant level of clinical complexity and frailty.

Our literature review suggests that vortioxetine shows an efficacy profile different from other antidepressants. It is probably less effective than many other agents in reducing mood symptoms, but it seems to have unique properties on cognitive symptoms and negative symptoms, including emotional blunting [11]. Since a large part of hospitalized patients do not meet the criteria for severe depressive states (melancholia) and exhibit somewhat milder demoralization experiences close to adjustment disorders [154,155,156,157,158,159,160,161,162], wherein somatic symptoms, cognitive distortions and a weakening of attentive functions play a key role, vortioxetine could be much more tailored to these forms. On the other hand, melancholic patients are less likely to respond to vortioxetine and might need a more incisive kind of antidepressant, ideally, tricyclic antidepressants [163,164], provided that clinical conditions and access to TDM can safely allow for their employment. Thanks to its long half-life and strong serotonergic activity, vortioxetine also seems to be an option to cover the withdrawal symptoms of SSRIs. Eventually, as inferable from its very diversified pharmacodynamic profile, it seems to be of promising usefulness in patients that require a therapeutic switch for unacceptable behavioural toxicity, eminently SSRI-induced affective and motivational issues (including SSRI-induced sexual dysfunction).

All of the features above can give vortioxetine great momentum in CL psychiatry. Safety and tolerability are probably the main strengths of this drug [165,166,167]. The availability of pharmacokinetics and safety data relative to subjects with severe liver and kidney dysfunction provides the clinician with sound scientific evidence for its manageability in nephropathic and hepatopathic patients, for whom many psychotropics are often precluded or heavily restricted. The probable lack of significant effects on myocardium repolarization is also a considerable strength of this drug [168]. The frequency of electrolyte imbalances (namely hyponatremia) appears to be lower with vortioxetine than with SSRIs, although vortioxetine is still associated with a non-negligible amount of risk, especially when combined with sodium-wasting diuretics. Nonetheless, to implement the employment of vortioxetine as safely as possible, its most common side effects are to be carefully evaluated [169,170]. These appear to occur in two main clusters: gastrointestinal TEAEs (nausea, vomiting, Diarrhoea), which are type A dose-dependent reactions and can be severe in a minority of subjects, or hypersensitivity TEAE, which are mostly type B dose-independent reactions, oftentimes involving the integumentary system (pruritic symptoms, petechial rashes) and can also be severe. The former type of TEAE is predictable, tends to subside with time, is often treatable and does not always demand drug interruption, while the latter is unpredictable, could catch the clinician unawares and requires immediate drug discontinuation.

Vortioxetine seems to be linked to both favourable and unfavourable neurobehavioural side effects: on the one hand, it seems to promote cognitive and psychomotor performance, allegedly being one of the antidepressants with the mildest risk of diurnal sleepiness; on the other, vortioxetine seems to raise a non-negligible risk of mood lability, anxiety and agitation in a minority of patients. The effects on sleep structure seem to be similar to those of SSRIs, and the risk of a decline in sleep quality should always be sought.

A great advantage of vortioxetine compared to most SSRIs is its limited potential for pharmacokinetic interactions [8]. In the literature here reviewed, there was no evidence of significant inhibitory activity on any cytochrome p-450 isoenzyme, and the only remarkable interactions implicated pharmacodynamic mechanisms shared by all SSRIs, together with venlafaxine and duloxetine (increased risk of bleeding with antiplatelets or anticoagulants). Conversely, SSRIs (especially paroxetine, fluoxetine and fluvoxamine) are perpetrators of a host of interactions due to enzyme inhibition and subsequent victim drug-related overdosage toxicity.

The present work has several limitations. The largest part of these limitations is implied by the nature of the case series design (small sample, no inferential statistics allowed). Another important limitation is the lack of extant comparable data about vortioxetine use in hospitalized patients with high degrees of complexity and fragility. Furthermore, the adoption of strict inclusion criteria had a twofold consequence: on the one hand, it allowed the selection of particularly “high-risk” patients in which to assess safety and tolerability, but on the other, it might have led to a sample that does not mirror the largest part of clinical pictures encountered in daily medical practice. Another significant limitation was the relatively short observation time after vortioxetine introduction in some of our cases.

## Figures and Tables

**Table 1:** Personal data, medical comorbidities and surgery in included patients.

Patient	Age	Sex	Medical Comorbidity	Surgery during Hospitalization
P01	65	M	High-grade retroperitoneal sarcoma Pleuropulmonary infection Pulmonary embolism	En-bloc resection of sarcoma (including the head of pancreas, duodenum, right kidney, right adrenal gland and right colon)
P02	77	M	Complicated diverticulosis Chronic ischemic heart disease Atrial fibrillation COPD Chronic anemia Type 2 diabetes mellitus	Sigmoidectomy
P03	64	F	Advanced endometrial carcinoma Intestinal occlusion Multiple electrolyte imbalance	Decompressive colostomy
P04	77	M	Class 3 obesity Type 2 diabetes mellitus Systemic arterial hypertension Atrial fibrillation Respiratory insufficiency Burns (15% TBSA)	Multiple escharotomies and reconstructive procedures
P05	74	F	Chronic kidney disease Recurrent urinary infections Polymetastatic thyroid carcinoma	None
P06	73	M	Ischemic heart disease Atrial fibrillation Long QT Thigh sarcoma Type 2 diabetes mellitus	Thigh sarcoma excision (limb-sparing surgery)

M: male; F: female; COPD: chronic obstructive pulmonary disease; TBSA: total body surface area.

**Table 2:** Vortioxetine dose and co-prescribed psychotropic and non-psychotropic medications.

Patient	Vortioxetine Dose	Observation Length	Associated Psychotropics	Non-Psychiatric Pharmacotherapy
P01	5 mg	4 weeks	None	Bisoprolol Pantoprazole Enoxaparin Ondansetron
P02	5 mg	4 weeks	Delorazepam 0.5 mg/day	Pantoprazole Metoprolol Amiodarone Linagliptin Furosemide Umeclidinium Mesalazine Warfarin
P03	10 mg	1 week	Morphine 40 mg/day Buprenorphine Patch 70 mg/h Pregabalin 75 mg/night	Ondansetron Domperidone Chemotherapy (Doxorubicin and Cisplatin)
P04	10 mg	6 weeks	Melatonin 2 mg/night Pregabalin 150 mg/day	Pantoprazole Olmesartan Amlodipine Furosemide Amiodarone
P05	10 mg	1 week	Lorazepam 1 mg/night	Ursodeoxycholic acid Fondaparinux Metoclopramide Pantoprazole Levothyroxine
P06	10 mg	8 weeks	Divalproex 750 mg/day Delorazepam 0.5 mg/night	Metformin Ranolazine Zofenopril/HCTZ Pantoprazole Metoprolol Furosemide Rivaroxaban

**Table 3:** Efficacy and safety data.

Patient	CGI-S Baseline	CGI-S Follow-Up	CGI-I	CGI-EI	Safety Issues	Discharge
P01	4	3	2	1	None	Discharged to home
P02	3	2	2	5	None	Discharged to home
P03	4	4	4	13	None	Transferred to palliative care facility
P04	4	3	3	9	None	Transferred to intermediate care facility
P05	3	2	2	5	None	Discharged to home
P06	5	3	2	5	Nausea	Discharged to home

## Data Availability

The data presented in this study are available on request from the corresponding author.
